# Utilization of glycosaminoglycans by the human gut microbiota: participating bacteria and their enzymatic machineries

**DOI:** 10.1080/19490976.2022.2068367

**Published:** 2022-04-28

**Authors:** Parkash Singh Rawat, Ahkam Saddam Seyed Hameed, Xiangfeng Meng, Weifeng Liu

**Affiliations:** State Key Laboratory of Microbial Technology, Microbial Technology Institute, Shandong University, No.72 Binhai Road, Qingdao 266237, P. R. China

**Keywords:** Glycosaminoglycans, *Bacteroides*, Firmicutes, polysaccharide lyase, gut microbiota

## Abstract

Glycosaminoglycans (GAGs) are consistently present in the human colon in free forms and as part of proteoglycans. Their utilization is critical for the colonization and proliferation of gut bacteria and also the health of hosts. Hence, it is essential to determine the GAG-degrading members of the gut bacteria and their enzymatic machinery for GAG depolymerization. In this review, we have summarized the reported GAG utilizers from *Bacteroides* and presented their polysaccharide utilization loci (PUL) and related enzymatic machineries for the degradation of chondroitin and heparin/heparan sulfate. Although similar comprehensive knowledge of GAG degradation is not available for other gut phyla, we have specified recently isolated GAG degraders from gut Firmicutes and Proteobacteria, and analyzed their genomes for the presence of putative GAG PULs. Deciphering the precise GAG utilization mechanism for various phyla will augment our understanding of their effects on human health.

## Graphical abstract

**Figure:**
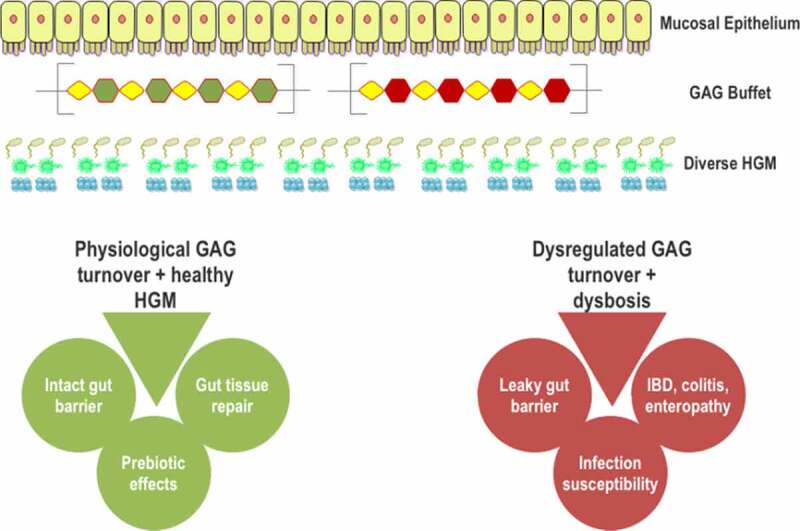

## Introduction

The human gut microbiota (HGM) is now considered to be one of the most metabolically active “organs” of the human host^1,2^ and contributes to human health in numerous ways.^3^ Remarkably, the HGM collectively catabolizes the indigestible fiber and several host-derived glycans to produce short-chain fatty acids (SCFA) for improving human health.^4–8^ The host colonic glycans, including mucosal carbohydrates, glycosaminoglycans (GAGs), and glycosphingolipid, provide a continuous source of nutrition for the HGM.^9–11^ In contrast, dietary carbohydrates can vary in types and quantity based on the diet and timings of the meal.^3^

Due to the constant presence of host glycans, such as GAGs, their metabolism by the HGM plays critical roles in the colonization of the gut and in modulating the composition of HGM, and thus provides significant benefits to the gut bacteria and the host. Utilization of GAGs, especially chondroitin sulfate (CS) and heparin has been found to be indispensable for various members of the HGM. Cheng and Salyers, 1995^12^ have shown that a *Bacteroides thetaiotaomicron* mutant deficient for CS and heparin utilization was incapable of colonizing the gut of germ-free mice, demonstrating the importance of GAGs as a carbon substrate for the gut microbiome and hence their colonization. In another study, it was shown that wild type *B. thetaiotaomicron* out-populated a pre-colonized CS and heparan sulfate (HS) utilization deficient *B. thetaiotaomicron* mutant strain within 4 d of gut colonization in the mice, illustrating the advantage of GAG utilization.^13^ It has also been reported that foraging on intestinal glycans helps in the colonization and metabolic niche development of Firmicutes,^14^ *Akkermansia*,^15^ and Actinobacteria and Proteobacteria.^16^ In addition, several pathogens are also reported to utilize mucosal and epithelial surface glycans for invasion.^17–19^ The HGM can, in principle, out-compete these pathogens through host-glycan utilization and hence prevent their invasion.^20–23^ However, excess foraging of host-derived polysaccharides has shown detrimental effects on the colonic epithelium.^24,25^ For example, the intestinal mucosa was found to be more susceptible to pathogenic infection when the microbiota showed a heightened mucus consumption.^24^ Similarly, Liao et al., 2017^25^ showed that excessive foraging of CS in the gut could induce various inflammatory and opportunistic infections. Furthermore, Lee et al., 2009^26^ demonstrated that CS and hyaluronic acid (HA) degradation by the mice gut microbiome might be linked to the induction and propagation of colitis. Glucosamine and galactosamine, the degradation products of HA and CS, respectively, were found to be cytotoxic to the intestinal cells.^26^ Therefore, a balanced host glycan utilization is necessary for a healthy gut. GAGs are one of the most important host glycans and are abundantly present in the intestine by continuously shedding from the gut epithelium.^9,27^ A detailed summary of GAG-degrading gut bacteria, their degradation mechanism, and regulation of the pathway would be highly helpful for understanding the interaction between host GAGs and the HGM. Our present review attempts to illustrate the current information of the host GAG degradation by the HGM.

## Structure of the host GAGs and their presence in the Gut

**Figure 1.**
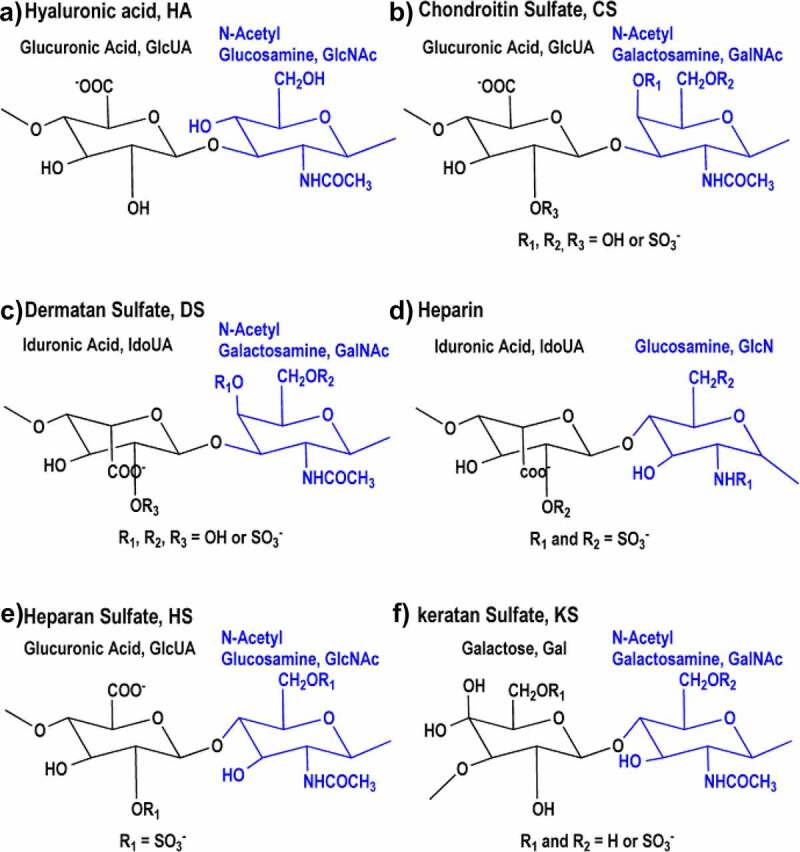
Structures of the repeat units in various GAGs. (A) HA is an unsulfated polymer with [−4)-β-GlcUA-(1-3)-β-GlcNAc-(1-] as the repeating unit. (B) CS is the polymer of sulfated [−4)-β-GlcUA-(1-3)-β-GalNAc-(1-]. Depending on the sulfation pattern, CS can be classified into different subtypes (CSA, CSC, CSD, and CSE). (C) DS composes of the repeating dimeric unit [−4)-α-IdoUA-(1-3)-β-GalNAc-(1-]. L-IdoUA is an epimer of D-GlcUA and the GalNAc residue is sulfated at different positions similar to CS. (D) The major disaccharide unit of heparin is [−4)-α-IdoUA-(1-4)-α-GlcN-(1-]. Iduronic acid is sulfated at O2 and the GlcN unit is N- and O6-sulfated. (E) The major disaccharide unit of HS is [−4)-α-GlcUA-(1-4)-β-GlcNAc-(1-]. The GlcNAc residue is generally sulfated at N2 with or without O6 sulfation. (F) In the typical KS, disaccharide subunits are composed of Gal and GlcNAc-6-sulfate. The Gal residues may or may not have O6 sulfation.

GAGs are negatively charged, amino sugar-containing polysaccharides found throughout the human body.^28^ Depending on the repeating disaccharide, they are categorized into HA, CS, dermatan sulfate (DS), heparin, HS, and keratan sulfate (KS) (Figure 1). Structurally, HA is the simplest of the GAGs. It is an unsulfated polymer composed of repeating disaccharide units of glucuronic acid (GlcUA) and N-acetyl glucosamine (GlcNAc) linked by (β1-3) and (β1-4) linkages, respectively. CS are also linear polymers composing of the repeating unit of [−4)-β-GlcUA-(1-3)-β-N-acetyl-galactosamine (GalNAc)-(1] with different sulfation patterns. The GalNAc residues of CS can be sulfated at O4 (CSA), O6 (CSC), or both (CSE). In CSD, O4 of GalNAc and O2 of GlcUA are both sulfated. The basic disaccharide unit of DS is composed of Iduronic acid (IdoUA) and GalNAc linked by (α1-3) and (β1-4) linkages. Heparin and HS are highly sulfated GAGs with repeating disaccharide unit comprising hexeuronic acid (IdoUA or GlcUA) and glucosamine (GlcN). Among GAGs, heparin and HS exhibit the highest structural heterogeneity. In heparin, the major hexeuronic acid residues are O2-sulfated-IdoUA, which are (α1-4) linked to GlcN mostly with sulfation at N2 and O6. In contrast, the major hexeuronic acid in HS is O2-sulfated-GlcUA which is (β1-4) linked to O6-sulfated-GlcNAc. Generally, heparin exhibits higher sulfation levels compared to HS. KS is the only GAG devoid of hexeuronic acid. The repeating unit of this polymer is composed of [−3)-β-Gal-(1-4)-β-GlcNAc6S-(1-]. A significant number of galactose residues are O6-sulfated. The enteric KS type, lactosaminoglycan, lacks sulfation at both the residues.^29^

HA is present only in free-form and not as a part of proteoglycans (PGs) in the gut, while other GAG types are present both as part of PGs, forming the extracellular matrices of almost all mammalian tissues, and free-form.^28^ Sulfated GAGs are highly distributed in the lamina propria, the basal lamina, and the lumen of the crypts of the colonic mucosa.^30^ HA is the most abundant GAG in the gut epithelium, with a content range of 0.82–0.95 μg/mg of the dry weight of intestinal tissue, followed by HS (0.37–0.5 μg/mg), DS (0.22–0.36 μg/mg), and CS (0.05 μg/mg).^31^ Although keratan sulfate (KS) is prominently present in non-gut tissues,^32^ a non-sulfated form called lactosaminoglycan is reported to be present in gastrointestinal tract mucins.^29^ Under physiological cellular turnover and various pathological conditions, GAGs from the mucosal surfaces are shed into the intestinal lumen.^9,27,33^ For example, Syndecans, a combination of heparin sulfate and chondroitin sulfate proteoglycan (CSPG), is significantly over-shed in colitis.^34^ Iozzo, 1987^9^ have shown that approximately 55% of newly synthesized heparin sulfate proteoglycans (HSPG) are secreted and not internalized by the cells.

## Host GAGs and the HGM – Physiological implications

The prevalence of GAGs throughout the human body and their structural complexity make them highly biologically active.^28^ Within the colon, their physiological role is further amplified due to their degradation by the HGM. As these degradation products are also bioactive, their disordered utilization could be detrimental.^24,25^ Also, the total contents of individual GAGs have been shown to undergo alteration in inflammatory bowel disorders (IBDs).^30^ GAG-PGs are essential for gut tissue development during embryogenesis.^35^ Various HSPGs and CSPGs are involved in the modulation of cell shape, cell motility, contact inhibition, and intestinal morphogenesis.^36–38^ Dysregulation in the production and binding of these proteoglycans to the basement membrane has shown to be oncogenic in the colon.^38^ Mice deficient in the enzyme required for the initiation of HS sulfation showed altered colon histology and an increased rate of colonic epithelium apoptosis.^39^ Another important function of GAG-PGs is the maintenance of the gut barrier function.^40^ Membrane homeostasis is shown to be achieved by the modulation of epithelial regeneration through HSPGs^41^ and HA.^42^ The loss of barrier function is directly associated with the protein leaking enteropathy,^43^ colitis, and IBD.^41,44^

Similar to other surface glycans, GAGs and PGs can act as signaling molecules in the intestine. For example, polymeric HA can bind intestinal cells by CD44 and RHAMM (receptors for HA mediated motility) receptors, which are shown to be crucial for cellular turnover of HA and intestinal cell migration, respectively.^45^ In contrast, fragmented HA can bind Toll-like receptors 2 and 4 to induce proinflammatory cytokines.^45^ Surface HS has been reported to facilitate the binding of Wnt to the intestinal epithelial cells (IECs), which promotes intestinal crypts regeneration.^46^ Exogenous HS and heparin disaccharides have been shown to inhibit spontaneous as well as TNF-α-induced IL-8 and IL-1β secretion from IECs.^47^ A combination of HSPG with CSPG has been shown to be essential for the association of angiogenin with intestinal cells, indicating their roles in angiogenesis.^48^ Keratan sulfates are also involved in various signaling pathways throughout the body.^32^ However, its role in the gut is not very well understood, except that lactosaminoglycan has been shown as the receptor for *E. coli* type I enterotoxin in intestinal epithelial cells.^49^

Oral administration of various GAGs has also shown to be beneficial. Clinical trials have found that oral consumption of CS was beneficial in canine IBD^50^ and in minimizing IBD relapses in humans.^51^ Daily doses of pharmaceutical-grade CS have also been shown to be effective in managing arthritic knee pain and associated functions.^52^ A pharmacokinetic study suggests that these pain-relief effects could stem from the accumulation of CS or its degradation products in the gut or the liver.^53^ Prebiotic CS was also effective in reducing type 2 diabetes mellitus symptoms in the murine model.^54^ Dietary CS increased the sulfate-reducing bacteria, *Desulfovibrio piger* in the mice microbiome, which in turn enhanced the levels of the signaling molecule, hydrogen sulfide (H_2_S), in the colon. The elevated H_2_S levels improved glucagon-like peptide-1 and insulin secretion, glucose clearance, and reduced food consumption in diabetic mice.^54^ In murine models, CS disaccharides altered the microbiota composition and increased the prevalence of *Bacteroides acidifaciens*.^6^ The *B. acidifaciens* strain has been shown to prevent pathogenic colonization in the mice gut *via* inducing IgA production^55^ and prevent mice obesity *via* improved insulin sensitivity.^56^ Oral administration of other GAGs has shown beneficial effects for the host as well. Dietary DS, heparosan, and KS modulate the gut microbiota by increasing the beneficial *Lactobacillus* bacteria.^57,58^ HS administration, in the form of its analog enoxaparin, has shown improvement in mucosal healing using the mice colitis model.^59^ Dietary HS is also reported to recover the renal functions in the nephrectomized rats.^60^ Finally, low molecular weight HA was found to be effective in reducing the membrane permeability associated with colitis.^61^ The same study showed the protective effect of HA against *Citrobacter* infection. As these GAGs are shown not to be metabolized by the host, the beneficial effects of oral administration of GAGs could be attributed to the modulation of gut microbiota and the metabolites produced by their microbial degradation.

## Degradation of GAGs by the HGM – Participating bacteria

The physiological functions of GAGs and the beneficial effects of their oral administration make the study of bidirectional interaction of gut microbiota and GAGs critical. The continuous supply of GAGs in the intestinal lumen can provide a constant source of nutrition to the HGM.^62^ Particularly, various have been identified as degraders of GAGs. Although bacteria for the GAG degradation in other phyla are less recognized, a substantial number of non-Bacteroidetes gut bacteria, including Firmicutes and Proteobacteria, have recently been shown to catabolize GAGs. These gut bacteria employ various numbers and types of GAG-catabolism carbohydrate active enzymes (CAZymes) in their genomes for GAG degradation.

The complex structures of GAGs present a challenge in their breakdown by the members of the HGM and demand extensive catalytic competence from the gut commensals. Hence, different groups of colonic bacteria might have evolved a unique set of strategies to assimilate these glycans. Since GAG degradation is associated with gut microbiota colonization, gut disorders, and prebiotic effects, it is vital to identify the gut microorganisms and their strategies employed for the degradation of GAGs to understand the mutualistic effects of gut microbiota and host.

### Bacteroides

Bacteroidetes represent the second-largest bacterial phylum in the HGM.^63^ These Gram-negative bacteria are capable of catabolizing structurally diverse polysaccharides, including GAGs.^64,65^ This ability is attributed to the abundance of CAZymes in their genomes.^66^ *B. thetaiotaomicron* VPI-5482, the most studied gut bacterium, has been reported to be capable of utilizing various GAGs, including CS, DS, HA, and HS.^67–69^ Its HS degrading activity was reported to be induced by HS.^70^ Various other Bacteroides, including *B. uniformis* VPI 0061;^64^ *B. stercoris* HJ-15;^71^ *B. thetaiotaomicron* WAL2926;^72^ *B. ovatus* ATCC 8483;^73^ *B. cellulosilyticus* WΗ2,^74^ *B. caccae* ATCC 43185, *B. cellulosilyticus* DSM 14838, and *B. intestinalis* DSM 17393;^75^ *B. xylanisolvens* G25;^76^ *B. clarus* DSM 22519 *and B. paurosaccharolyticus* JCM 15092;^77^ and *B. eggerthii* DSM 20697^69^ have also been shown to be capable of assimilating CS. Most CS-degrading *Bacteroides* strains are also shown to be DS and HA utilizers.^69,71,72^

Similarly, heparin/HS chains are also assimilated by various gut *Bacteroides*, namely, *B. thetaiotaomicron* VPI-5482, *B. ovatus* ATCC 8483, and *B. eggerthii* DSM 20697;^64^ *B. uniformis;*^78^ *B. stercoris* HJ-15;^71^ *B. thetaiotaomicron* WAL2926;^79^ *B. ovatus* ATCC 8483;^73^ *B. cellulosilyticus* WH2;^80^ and *B. cellulosilyticus* B19, *B. ovatus* A2, and *B. xylanisolvens* G25.^81^ Currently, the degradation of KS or lactosaminoglycan by the gut flora has not been reported. However, oral administration of KS has been shown to remarkably modulate gut microbiome of the mice, indicating that KS degraders are probably present in the gut.^58^

### Non-Bacteroidetes bacteria

Unlike *Bacteroides*, GAGs-degradation ability is not widely prevalent in other gut phyla. Salyers et al., 1997^82^ showed that a 154 fecal strains of Firmicutes and *Bifidobacteria* (Actinobacteria) failed to ferment CS, HA, and heparin. These strains could not utilize the monomeric sugar in these GAGs (D-galactosamine) as well. In another extensive study, 239 strains of *Bifidobacterium* were shown to be incapable of utilizing heparin, CS, hyaluronan, and polygalacturonate.^83^

*Streptococcus intermedius* UNS 35, an oral microbiota commensal, was the first Firmicute that showed GAG (CSA and CSC) utilization.^84^ Recently, a few more Firmicutes, *Hungatella hathewayi* R4;^76^ *Faecalibacterium prausnitzii* DSM 17677;^24^ *Enterococcus faecium* H57 and H59, *Lactobacillus casei* ATCC 393, and *L. pantheris* NBRC 106106^77^ have been demonstrated to be CS utilizers. These *E. faecium* strains were also able to degrade HA.^77^ In addition, *Proteus vulgaris*, which is a Proteobacteria and is considered part of the healthy gut microbiota,^85^ is known to harbor two well-characterized chondroitinases.^86–88^ The chondroitinases ABC of *P. vulgaris* can also depolymerize dermatan sulfate.^86^ Moreover, the chondroitin lyase activity was found in *Victivallis vadensis* ATCC BAA-548, a member of a newly discovered gut phylum *Lentisphaerae*.^80^ Compared to the other GAGs, heparin/HS has been shown to be catabolized by a larger number of non-Bacteroidetes gut bacteria. These include *E. faecium* strains H57 and H59, *Enterococcus faecalis* ATCC 19433, *Lactobacillus animalis* ATCC 35046, *L. casei* ATCC 393, *L. rhamnosus* ATCC 8530, *L. pantheris* NBRC 106106, *L. paracasei* JCM 8130, and *L. rhamnosus* Lc705.^77^

## The enzymatic machinery of GAG degradation in *Bacteroides*

The genetic loci responsible for polysaccharide degradation in Bacteroidetes are termed as polysaccharide utilization loci (PUL), which generally encode the entire enzymatic machinery required to catabolize a specific glycan. Proteins required for the pathway regulation, polysaccharide binding at the bacterial surface, incomplete initial degradation of the glycan, import of the oligosaccharides, and the complete breakdown of the saccharides are all encoded in glycan-specific PULs.^89^ In rare cases, an enzyme from a distant genetic location could be involved. For example, a sulfatase, BT1596 (S1_9), required for both CS and HS degradation in *B. thetaiotaomicron* is present remotely from their PULs.^90^

The starch utilization PUL (Sus) was the first completely elucidated PUL in *Bacteroides*. The genes in the Sus-PUL and their corresponding proteins in *B. thetaiotaomicron* are well characterized.^91–94^ For a detailed reading of the Sus operon, we direct readers to the excellent review by Flint et al., 2016.^95^ Briefly, SusD was determined to be a surface localized starch-binding protein, which complexes with SusC, a TonB-dependent porin, to mediate the transport of oligosaccharides released by a surface amylase, SusG. The presence of SusC-like and SusD-like genes is considered to be the signature of Bacteroidetes PULs.^89^ However, in minority cases, SusC/D is replaced by other types of transporters, such as major facilitator superfamily (MFS) proteins and ABC transporters. Recently, the presence of CAZyme gene clusters, containing a transcription regulator gene, a transporter gene, and a CAZyme is now considered as the definitive marker of a PUL.^89,96^

The GAG-PULs in most *Bacteroides* are shown to be inducible by specific GAGs; however, they do not show catabolite repression in the presence of glucose.^75,97^ Similar to the starch PUL, the GAG binding to the *Bacteroides* cell surface is facilitated by the homologs of SusD, while SusC homologs import GAGs to the periplasm. The GAG-specific PULs possess a set of enzymes called polysaccharide lyases (PLs) for the decomposition of polysaccharides. These proteins degrade the polysaccharides containing hexeuronic (UA) residues to disaccharides with unsaturated hexeuronic acid residue *via* a β-elimination mechanism.^98^ The liberated GAG-disaccharides are further degraded by glycoside hydrolase family 88 (GH88) enzymes to their respective monomers. In addition, the GAG-PULs include sulfatases for the desulfation of GAG polysaccharides and the liberated GAG-disaccharides.^99^ The characterized GAG-specific PULs and their homologous clusters in various *Bacteroides* also include hybrid two-component systems (HTCS) for transcription regulation of PUL genes.^75,97,99^ The PUL and mechanistic details of the CS and HS degradation by *B. thetaiotaomicron* are presented below to elucidate the *Bacteroides* enzymatic machinery for the degradation of GAGs.

### CS degradation machinery of *B. thetaiotaomicron*

**Figure 2.**
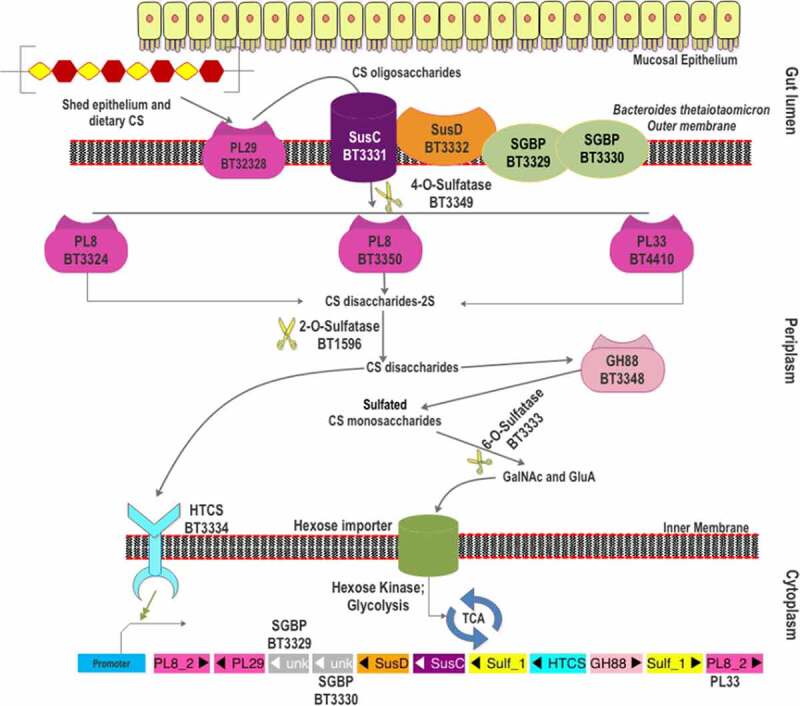
The scheme of chondroitin sulfate degradation by *Bacteroides thetaiotaomicron* VPI-5482. Turnover of gut epithelium and the diet are the two sources of CS in the gut. The surface-localized BT3328-PL29 dissimilates extracellular CS to produce oligomers, which are bound by SGBPs: BT3329, BT3330, and BT3332 (SusD-like), and internalized by BT3331-SusC-like transporter. These oligomers are processed by three periplasmic PLs (BT3324-PL8, BT3350-PL8, and BT4410-PL33) and BT3349-4-O-sulfatase (S1_27). The disaccharide products of these CS lyases are desulfated by BT1596 (S1_9) at the 2-O position. The GH88 Δ-4,5-unsaturated β-glucuronyl hydrolase (BT3348) processes these disaccharides to hexuronic acid and GalNAc or GalNAc6S monomers. BT3333-sulfatase (S1_15) removes the 6-O-Sulfate group from the GalNAc6S residues if required. All the unsulfated monomers are imported *via* the inner membrane into the cytoplasm for utilization. The HTCS protein BT3334 is activated by the BT1596 (S1_9) product.

Early in the 1990s, it was found that CS assimilation capability of *B. thetaiotaomicron* VPI-5482 was essential for the successful intestinal colonization of this strain.^12,100^ Afterward, its CS-specific PUL was identified by transcription profiling and has been well characterized (Figure 2).^67^ The CS utilization locus was also shown to be involved in the degradation of DS and HA.^69,101^ The CS-PUL of *B. thetaiotaomicron* VPI-5482 extends from *bt3324 to bt3350*, together with the distal *bt4410*.^67,97^ Similar to many other PULs in *Bacteroides*, the CS-PUL contains a SusD-like cell surface glycan-binding protein (SGBP) (BT3331) and a SusC-like TonB-dependent transporter (BT3332). Other surface proteins, BT3329 and BT3330, are also proven to be SGBPs.^69^ Within the PUL, four PLs are encoded, *viz*. BT3324, BT3328, BT3350, and the distal BT4410. Among them, BT3324 and BT3350 belong to polysaccharide lyase family 8 (PL8), whereas BT3328 and BT4410 belong to PL29 and PL33 families, respectively.^80,101,102^ A GH88 protein (BT3348) is also located in the CS-PUL for the breakdown of unsaturated disaccharides generated by PLs. As CS is a sulfated GAG, two sulfatases [BT3333 (S1_15) and BT3349 (S1_27)] in the PUL and another distant sulfatase [BT1596 (S1_9)] are required for the complete CS breakdown. BT3334, an HTCS regulator in the PUL, has been shown to regulate the entire CS degradation machinery.^97^

The first step in CS depolymerization by *B. thetaiotaomicron* was initially considered to be the desulfation of the polymer in the periplasm. However, Ndeh et al., 2018^101^ showed the presence of a surface-localized polysaccharide lyase (BT3328), which was identified as the founding member of the PL29 family. BT3328-PL29 was shown to cleave CS, DS, and HA through endolytic β-elimination. Product analysis revealed that a series of unsaturated oligosaccharides, such as disaccharide ΔUA-GlcNAc (ΔUA: unsaturated hexeuronic acid), tetrasaccharide ΔUA-GlcNAc-GlcUA-GlcNAc, hexasaccharide ΔUA-(GlcNAc-GlcUA)_2_-GlcNAc, and octasaccharide ΔUA-(GlcNAc-GlcUA)_3_-GlcNAc were produced by BT3328-PL29 from HA degradation.^101^ The surface localization of BT3328-PL29 indicates that the CS polymeric chains are endolytically cleaved by BT3328-PL29 to initiate the depolymerization. Notably, the extracellular CS degradation was not completely abolished in the ΔBT3328-PL29 mutant strain,^101^ suggesting the presence of other chondroitin lyases on the bacterial surface. An additional predicted chondroitin lyase (BT2964) in the *B. thetaiotaomicron* VPI-5482 genome could be the candidate PL for the cell surface degradation of CS.

The liberated CS oligomers are captured by three SGBPs: BT3331 (Sus-D like), BT3329 (SGBP), and BT3330 (SGBP).^69^ Binding studies showed that BT3331-SusD could significantly bind CSA only. In contrast, BT3329-SGBP could bind more substrates with the order of affinity (*K_a_*): CSC> DS> CSA> HA. For BT3330-SGBP, the affinity for CSA was found to be the highest followed by DS, HA, and CSC. Overall, compared to BT3330-SGBP and BT3331-SusD, BT3329-SGBP showed a higher affinity for each substrate and could accommodate smaller oligosaccharides with 10 degrees of polymerization (DP) and more varied sulfation patterns. In contrast, BT3330-SGBP could not accommodate CS chains of 10 DP, which might indicate its role in binding longer oligosaccharides. BT3331-SusD showed two folds higher affinity for CSA than BT3330-SGBP.^69^ Despite its limited substrate specificity, BT3331-SusD appears to be important as the inactivation of BT3331 abolishes the CS internalization^103^ and the expression of other CS-PUL genes.^97^ However, the functional roles of BT3329-SGBP and BT3330-SGBP have not been evaluated by gene deletion studies. SusD homologs are known to interact with SusC-homologs to form a “pedal-bin” assembly that allows the import of substrate glycans.^104^ This assembly facilitates the import of CS/DS/HA oligomers into the periplasmic space *via* a TonB-dependent importer. Protein interaction experiments reveal that the two SGBPs (BT3329 and BT3330) also interact with BT3328-PL29 *in vivo* and probably with BT3332-SusC,^69^ indicating their similar roles as that of SusD homologs. In the periplasm, these oligomers are desulfated at 4-O’ position by an endosulfatase, BT3349-4-O-sulfatase (S1_27),^69^ which shows high specificity toward 4-O-sulfate groups in the galactosamine residue, irrespective of sulfation at 6-O’ position.^90^ Though the 4-O-desulfation activity does not seem to be essential for the activity of the periplasmic PLs, removal of the 4-O-sulfate group shows an enhanced activity of BT4410-PL33 on CS oligosaccharides.^69^ Whether the 4-O-desulfation occurs before or after the action of periplasmic PLs is not yet known. However, the activity of BT3349-4-O-sulfatase (S1_27) is essential for producing 4-O-desulfated disaccharides as ligand/substrate of downstream BT3334-HTCS and BT3348-GH88, which cannot accept 4-O-sulfated disaccharides.^97^ The CS oligomers are depolymerized by the three periplasmic PLs (BT3324-PL8, BT3350-PL8, and BT4410-PL33) to release unsaturated CS disaccharides (CSΔdi).^69,97^ Although the three PLs are induced simultaneously, they show different catalytic efficiency, substrate specificity, and mode of action. BT3324 is shown to be a PL8 exolyase, with the preference of CSA over CSC and a low activity toward HA.^69,105^ In contrast, BT3350 is an endo-acting PL8 with similar substrate specificities but a higher specific activity than BT3324-PL8.^69,97,105^ Both PL8s are also active on DS. The third CS lyase, BT4410, has been assigned to the PL33 family based on sequence identity and the differences in substrate specificity.^80^ Compared to BT3324-PL8 and BT3350-PL8, BT4410-PL33 shows the lowest catalytic efficiency (*k_cat_/K_m_*) toward CS and DS.^69,97^ As its catalytic efficiency toward HA is higher than the other periplasmic PLs, it is designated as a hyaluronidase.

After the action of periplasmic PLs, an exolytic Δ4,5-hexuronate-2-O-sulfatase, BT1596 (S1_9), acts on the released CSΔdi to release the O2-sulfate groups from the hexeuronic acid residues.^90^ Notably, BT1596 (S1_9) is shown to be essential for the complete assimilation of both CS and HS, as the glycoside hydrolase in the downstream pathway is intolerant to the 2-O-sulfation of disaccharides. Once being desulfated at the 2-O position, the CSΔdi are further degraded by a periplasmic GH88 Δ-4,5-unsaturated β-glucuronyl hydrolase (BT3348) into 5-keto-4‐deoxyuronate and GalNAc monosaccharides. It is shown to act on both unsulfated and 6-O-sulfated disaccharides (CSΔdi6S), whereas 2-O- (CSΔdi2S) and 4-O-sulfated disaccharides (CSΔdi4S) are not processed.^97^ Specifically, BT3348-GH88 binds the unsulfated disaccharide about four times stronger than CSΔdi6S; however, the *k_cat_* for CSΔdi6S is about twice compared to the unsulfated disaccharide.^90^ Finally, the released GalNAc6S residues (for CSC and CSE) undergo 6-O-desulfation by the exosulfatase BT3333 (S1_15).^69,90^ As the PLs, sulfatase, and GH88 in the CS-PUL show a broad substrate specificity, the PUL *bt3324-bt3350* is also involved in the utilization of DS and HA.^67,69^

**Figure 3.**
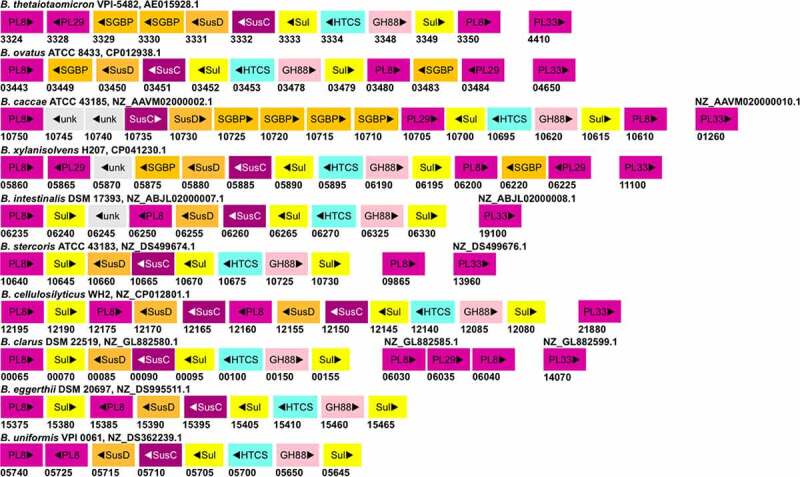
The gene organization of the CS-PUL in various gut *Bacteroides*. The CS-PUL in *B. thetaiotaomicron* spans from *bt3324-bt3350*, and a distant *bt4410* gene was also involved in CS degradation. PLs (purple), SusD and SGBP (orange), SusC (plum), GH88 (pink), sulfatase (yellow), and HTCS (cyan) encoded in PULs are indicated. Genome accession numbers are presented at the top of each *Bacteroides* species, and gene numbers of various genes are provided below each gene.

CS-PULs are also predicted in various other *Bacteroides* (Figure 3). Among these, the CS-PUL in *B. cellulosilyticus* WH2 (*Bcellwh2_RS12080-12,195*) has been characterized by transcriptional profiling.^74^ Similarly, the orthologous gene cluster in *B. ovatus* ATCC 8483 (*Bovatus_RS03443-03484*) has been shown to be induced in the presence of CS.^73^ Other *Bacteroides* CS-PULs have not been confirmed by either transcriptomic or biochemical studies yet. In *B. ovatus*, the *bt3329-sgbp* and *bt3328-pl29* homologous genes are present next to the *bt3350-pl8* homolog rather than next to the *bt3324-pl8* homolog. In *B. caccae* CS-PUL, there are insertions of two additional copies of *bt3329-sgbp* homologs before the *bt3330-sgbp* homolog, and thus in total, four SGBPs are encoded in the PUL. Whether these proteins have a redundant function remains to be studied. The number and position of SGBPs in CS-PUL of other *Bacteroides* strains also vary. In *B. intestinalis, B. eggerthii*, *B.* *cellulosilyticus*, and *B. stercoris* CS-PULs, no homolog of SGBPs is found. Compared to *B. thetaiotaomicron* VPI-5842, the PUL in *B. xylanisolvens* has an additional PL29 encoding gene. In contrast, *B. intestinalis, B. stercoris, B. cellulosilyticus, B. eggerthii*, and *B. uniformis* contain no PL29 homolog in their genome. The distant PL33 are encoded in most of these *Bacteroides* strains except *B. eggerthii* and *B. uniformis*. Similar to PL33, one PL8 gene of *B. stercoris* ATCC 43183 is located at a distant locus from its CS-PUL. In *B. clarus* DSM 22519, four PLs (two PL8, PL29, and PL33) genes are distantly located. Similar to *B. thetaiotaomicron* VPI-5482 BT1596, *B.* *ovatus, B.* *caccae, B.* *xylanisolvens*, and *B. uniformis* also contain a distantly located sulfatase gene. In contrast, the homologs of BT1596 (S1_9) are found to be encoded within the CS-PULs of *B. intestinalis, B. stercoris, B. cellulosilyticus, B. clarus*, and *B. eggerthii*. All these *Bacteroides* strains have been shown to degrade CS, suggesting that their CS-PULs are functional.

### Heparin/HS degradation machinery of *B. thetaiotaomicron*

**Figure 4.**
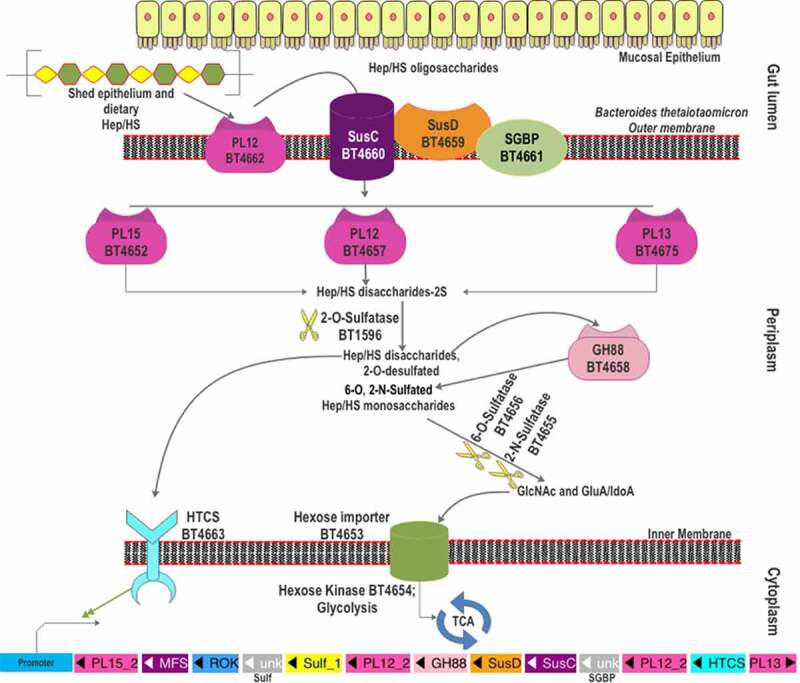
The scheme of heparin and HS degradation by *Bacteroides thetaiotaomicron* VPI-5482. Heparin/HS shed from epithelial cell line and acquired from the diet enter the gut lumen. A surface HS lyase (BT4662-PL12) depolymerizes these glycans into oligosaccharides, which are then internalized by BT4660-SusC *via* binding to BT4659-SusD and BT4661-SGBP. In the periplasm, three more heparin/HS lyases (BT4652-PL15, BT4657-PL12, and BT5475-PL13) cleave these oligomers to produce disaccharides. The disaccharides are desulfated at the 2-O position by BT1596 (S1_9). The product of sulfatase BT1596 is the substrate of the GH88 Δ-4,5-unsaturated β-glucuronyl hydrolase BT4658 and the activator ligand of HTCS protein. The monosaccharides liberated by GH88 are finally desulfated by BT4656-6-O-sulfatase (S1_11) and a yet unknown 3-O-sulfatase. The unsulfated monomers are imported into the cytoplasm for further metabolism. Hep: heparin.

Upon the induction by heparin and HS, the PUL spanning from *bt4652* to *bt4675* (Figure 4) was found to be upregulated in *B. thetaiotaomicron* VPI-5482.^67^ Various proteins encoded in this PUL have been comprehensively studied.^99^ One surface-localized PL, BT4662-PL12 and three periplasmic PLs, BT4652-PL15, BT4657-PL12, and BT4675-PL13, are found in this PUL. BT4661-SGBP and BT4659-SusD are encoded for glycan binding, while BT4660 is the SusC-like TonB-dependent transporter. Similar to the *B. thetaiotaomicron* CS-PUL, the distal BT1596-sulfatase (S1_9) is also involved in 2-O-desulfation in the process of HS decomposition.^90^ For 6-O-desulfation, BT4656 (S1_11) is required, while BT4655 is predicted as the 2-N-sulfatase based on functional analogy. A GH88, Δ-4,5-unsaturated β-glucuronyl hydrolase (BT4658), and an HTCS regulator (BT4663) are also found in the HS-PUL. In addition, BT4653 and BT4654 are predicted to be an inner-membrane sugar transporter and a cytoplasmic sugar kinase, respectively.

Heparin/HS glycan acquisition is achieved by BT4661-SGBP and BT4659-SusD homolog, which show physical association on the outer membrane.^99^ BT4661-SGBP deletion mutant did not show any growth defects on HS, whereas BT4659-SusD deletion mutant displayed a 30-h lag in growth on HS. However, both deletion mutants showed growth lag when grown on heparin oligosaccharides indicating their roles in the import of heparin/HS oligosaccharides.^99^ The two proteins also show contrasting binding affinities to HS and its oligosaccharides. BT4659-SusD shows a 20-fold lower *K_d_* on HS than that of BT4661-SGBP. In contrast, the heparin oligosaccharides, up to 10 DP, could only bind to BT4661-SGBP. Hence, it is assumed that the HS polymers bind to BT4659-SusD, while BT4661-SGBP binds the heparin/HS oligosaccharide. The bound polymer is firstly endolytically cleaved by the only surface-localized BT4662-PL12 to oligomers which are imported into the periplasm by the SusC-like transporter BT4660 and further degraded by three periplasmic PLs. BT4662-PL12 showed the highest activity on HS (100%), followed by heparin (40%) and unsulfated heparin (15%). In addition, BT4662-PL12 was found to be inactive on heparin oligosaccharides of DP 4 to 10, suggesting that it prefers longer chains for decomposition. Among the four PLs, BT4662-PL12 was found to be the least catalytically efficient (*k_cat_/K_m_*) on heparin and HS. However, it was more active on unsulfated heparin than BT4652-PL15 and BT4675-PL13.

Though it is the only surface HS lyase in the PUL, the BT4662-PL12 deletion mutant displayed only a marginal effect on the heparin/HS utilization. The authors attributed this to the presence of lower DP HS chains, which were bound by BT4661-SGBP and imported into the bacteria.^99^ In contrast, the single knockout mutants of BT4652-PL15 and BT4675-PL13 showed significant growth defects on heparin and to a lesser extent on HS. However, the ΔBT4657-PL12 mutant showed a delayed growth of >20 h on HS but not on heparin, indicating a significant role of BT4657-PL12 for HS degradation. The authors found that the combined deletion of BT4652-PL15 with one of the other two periplasmic PLs demonstrated remarkable growth defects. In particular, the Δ4652/Δ4657 double mutant showed almost no growth on HS and a >20 h growth lag on heparin, suggesting a critical role of periplasmic PL12 and PL15 for heparin/HS utilization. Similarly, the Δ4652/Δ4675 double mutant showed a ~30 h growth lag on heparin and less than half growth level on HS.

Degradation product analysis showed that BT4657-PL12 and BT4675-PL13 are endolyases, while BT4675-PL15 is an exoprocessive enzyme.^99^ The authors also analyzed the kinetics of the three PLs using highly sulfated heparin, moderately sulfated HS, and unsulfated heparin as substrates. End-point data suggest that BT4657-PL12 was least active on heparin and showed higher activity against HS and unsulfated heparin. In contrast, BT4675-PL13 showed significant activity on heparin while it was inactive on unsulfated heparin, indicating that the sulfation was compulsory for its activity. BT4652-PL15 was active on all three substrates but with higher activity on heparin than unsulfated heparin, suggesting a critical but not compulsory role of sulfation on activity. Kinetic analysis showed that the catalytic efficiencies (*k_cat_/K_m_*) on HS follow the order of BT4657-PL12≈ BT4675-PL13≫ BT4652-PL15, on heparin: BT4675-PL13> BT4657-PL12≫ BT4652-PL15, and on unsulfated heparin: BT4657-PL12≫ BT4652-PL15. Hence, BT4657-PL12 can effectively target unsulfated and low-sulfated regions of heparin/HS, while BT4675-PL13 can process the heavily sulfated regions of the polysaccharide chain. Authors argue that the oligosaccharides produced by the action of these two endolyases (BT4675-PL13 and BT4657-PL12) are digested further by BT4652-PL15 to produce the heparin/HS disaccharides, explaining the importance of it for heparin/HS utilization.^99^ The complementary activities of the three periplasmic PLs might have assisted the bacteria in achieving efficient utilization of heparin/HS with substantial structural variations, especially the heterogeneous degree of sulfation.

As in the case of CS degradation, the disaccharides produced from the degradation of HS by the PLs cannot be utilized by the bacteria prior to 2-O-desulfation, which is catalyzed by 2-O-sulfatase, BT1596 (S1_9).^90^ BT1596-2-O-sulfatase thus represents the connecting link for the CS and heparin/HS degradation pathways. Similar to the CS-PUL, BT1596 (S1_9) is distant from the HS-PUL. The 2-O-desulfated HS disaccharides (HSΔdi) are then degraded by GH88 Δ-4,5-unsaturated β-glucuronyl hydrolase (BT4658), which is active on the disaccharides with N2 (HSΔdiNS) or O6 (HSΔdi6S) sulfation but not O2-sulfation (HSΔdiUA2S).^99^ The 2-O-desulfated disaccharides are also shown to be the ligand for the HTCS sensor BT4663. Although the growth of BT1596 (S1_9) knockout strains in HS or CS minimal media has not been examined, deletion of the *bt0238* gene (encoding an anaerobic Sulfatase Maturating Enzyme, anSME) abolished the utilization of sulfated GAGs and mucins, suggesting the importance of desulfation in the GAG utilization.^12,13^ The insertional mutants of the *bt0238* gene were reported to be unable to colonize the gut of germ-free mice, signifying the importance of the CS and heparin/HS utilization pathways in the intestinal colonization for *B. thetaiotaomicron*.^100^ The N2 and O6-sulfated monosaccharide products, released by BT4658-GH88, are desulfated by BT4655 and BT4656 (S1_11), respectively. Although the biochemical characterization of BT4655 is not yet available, the ΔBT4655 mutant was shown to accumulate the N2-sulfated glucosamine in the media, implying its role in N2 desulfation.^99^ BT4656 (S1_11) is identified as an exo-O-sulfatase for the O6 desulfation from the GlcNS6S or GlcNAc6S.^90^ Occasionally, a portion of heparin/HS possesses 3-O-sulfation; however, sulfatase required to remove it is not yet characterized. A potential 3-O-sulfatase is BT1918 (S1_46), which has been characterized as GlcNAc3S,6S 3-O-sulfatase,^106^ however, transcription profiling of *B. thetaiotaomicron* on HS did not show an upregulation of *bt1918* gene.^67^ The desulfated monosaccharides are internalized to the cytoplasm by the putative hexose transporter, BT4653, and further phosphorylated by the hexokinase, BT4654. However, the ΔΒΤ4654 mutant did not show impaired growth on HS, indicating that the bacterium can use paralogous hexokinases to utilize these monosaccharides.^99^

**Figure 5.**
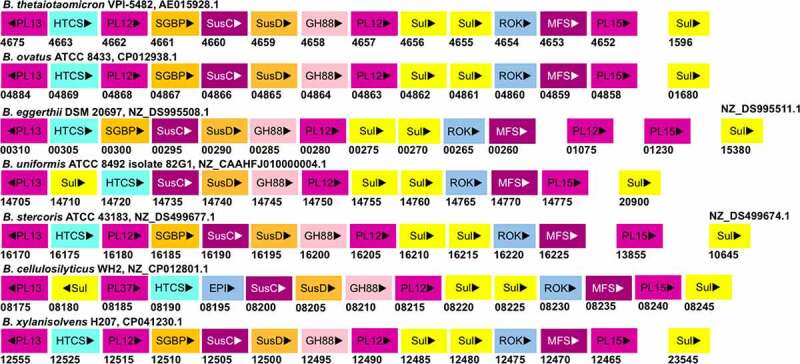
The gene organization of the Heparin/HS-PUL in various gut *Bacteroides*. The HS-PUL in *B. thetaiotaomicron* VPI-5482 spans from *bt4652-bt4675*. PLs (purple), SusD and SGBP (Orange), SusC and MFS (plum), GH88 (pink), sulfatase (yellow), HTCS (cyan), and ROK (blue) encoded in PULs are indicated. Genome accession numbers are presented at the top of each *Bacteroides* species and gene numbers of various genes are provided below each gene.

The heparin/HS-PUL is conserved among various gut *Bacteroides* (Figure 5). However, there are only three other species with genetically characterized heparin/HS-PULs. The PUL in *B. ovatus* ATCC 8483 (*bovatus_04856-04884*) shows a complete synteny to the *B. thetaiotaomicron* VPI-5482 PUL explained above.^67^ The other two genetically characterized PULs are *btheta7330_03329-03340* in *B. thetaiotaomicron* 7330 and *bcellwh2_01699-01710* in *B. cellulosilyticus* WH2.^74,107^ Among the *Bacteroides* species listed in Figure 5, all contain a PL15 gene, although some of them are located distant from their heparin/HS PUL. The gene coding for BT4662-PL12 homolog is absent in the heparin/HS PUL of *B. uniformis* and *B. cellulosilyticus* WH2, while it is present distantly to the PUL in *B. eggerthii*. However, a gene coding for a PL37 protein is present in the heparin/HS PUL of *B. cellulosilyticus* WH2 and the activity of this PL37 remain to be characterized. The PUL in *B. cellulosilyticus* WH2 has two additional sulfatases compared to the *B. thetaiotaomicron* VPI-5482 PUL, one of which is homologous to *bt1596-2O-sulfatase*. For the rest of the listed *Bacteroides* strains, the BT1596 (S1_9) homolog is present distant to the heparin/HS PUL. All these strains have been shown as heparin/HS degrader, indicating their heparin/HS PULs are functional.

### The regulation of GAG degradation in Bacteroides

It has been reported that *B. thetaiotaomicron* consumes CS and heparin/HS with a high priority without the repression by glucose and other polysaccharides.^108–110^ In *B. thetaiotaomicron* VPI-5482, both HS and CS PULs are regulated by a constitutively expressed HTCS regulator.^97,111^ Briefly, at higher GAG levels, the unsaturated disaccharide product of CS or heparin/HS degradation binds the HTCS regulator. The bound regulator upregulates the complete GAG-PUL, including the GH88 gene. At diminishing GAG levels and increased production of GH88 enzymes, the disaccharide products are driven away from the HTCS regulator by cleaving it to monosaccharides, thus resulting in a dynamic regulation of GAG-PUL genes. A conventional two-component sensor system utilizes a DNA-binding protein and a separate sensor-response protein to regulate gene transcriptions. However, in the HTCSs prevalent in the gut *Bacteroides* glycobiome, a single protein contains all domains, *i.e*., the histidine-kinase (HK) sensor domain, the phosphotransferase domains, the response regulator domains, and the DNA-binding, helix-turn-helix AraC domains (HTH_AraC).^112^ Hence, HTCS represents a novel adaptive strategy for glycan foraging in *Bacteroides*, in which an HTCS protein encoded by a single gene upregulates its adjacent PUL and can simultaneously repress PULs specific for utilization of other glycans.^113,114^ In the GAG utilization pathways, the HTCS regulators are located in the inner membrane.^69,111^ The binding of ligands to the periplasmic sensor domain of the HTCS dimerizes its cytoplasmic domains for autophosphorylation, which is relayed to the response regulator through the phosphotransferase domain. Finally, the DNA binding domain is activated through intramolecular phosphotransfer for upregulating the expression of PUL genes. In the CS and HS degradation pathways, unsaturated disaccharides, the products of PLs, are identified to be the ligands for the periplasmic HTCS sensor domains. In the CS degradation, unsulfated unsaturated chondroitin disaccharides (CSΔdi0S) and CSΔdi6S but not CSΔdi4S bind the HK sensor.^97^ As mentioned above, CS-4-O-sulfatase [BT3349 (S1_27)] removes the 4-O-sulfates from the unsaturated CS disaccharides, generating the required ligands for the HK sensor. In the HS pathway, sulfated HS GlcNS disaccharides (HSΔdiNS6S), unsaturated HS GlcNAc disaccharides (HSΔdiNAc6S) and their 6-O-desulfated forms (HSΔdiNS, and HSΔdiNAc) could bind the HK sensor domain.^111^ In both CS and HS degradation, BT1596 (S1_9) is required to remove 2-O-sulfates from hexuronic acid residues before the disaccharides can bind the HTCS sensor.^99^

Importantly, the HTCS sensor also provides a feedback mechanism for GAG utilization. The presence of the GAGs induces a transient rise in the transcripts of the PUL genes, thus generating a large number of unsaturated disaccharides.^97^ Interestingly, the HTCS and the GH88 proteins share the same substrate specificity in the GAG-PULs. However, the GH88 enzyme shows a higher *K_m_* than the HTCS, allowing the initial accumulation of PL-liberated unsaturated disaccharides and thus the rapid expression of the catabolic machinery, including the periplasmic PLs.^97,99^ As the GH88 is also induced, its heightened levels compete for the unsaturated disaccharides with the HTCS sensor, and hence, the expression of PUL is downregulated afterward. This novel strategy assures the appropriate expression of genes in the GAG-PULs and avoids an excessive expression of these proteins, which is probably important for ensuring the balanced degradation of host glycans. This mode of regulation may expand to other glycan utilization by *Bacteroides* strains but remains to be studied.

## The enzymatic machinery of GAG degradation in Non-Bacteroidetes

**Figure 6.**
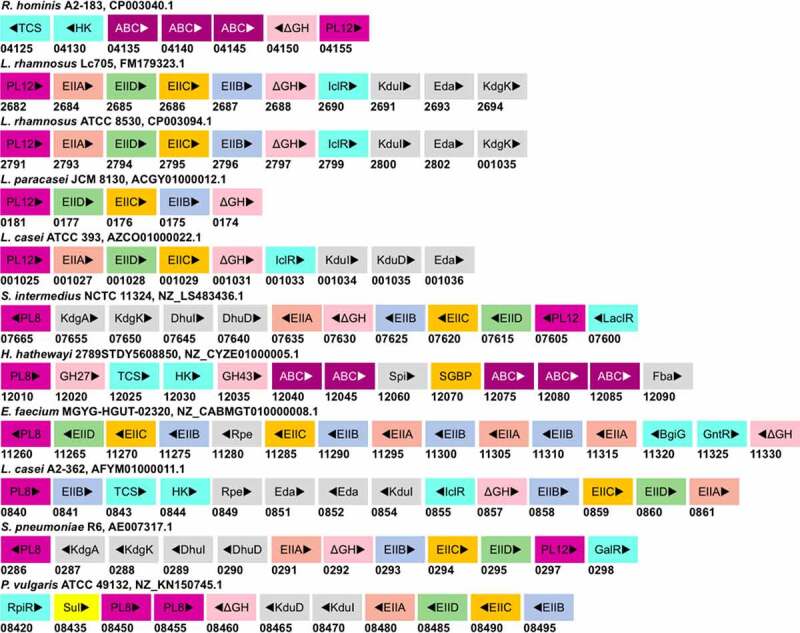
Putative GAG-utilization genomic loci in non-Bacteroidetes gut bacteria. Except for *Roseburia hominis* A2-183, other species have been shown to degrade GAGs. The heparin gpPUL in *Roseburia hominis* A2-183 is predicted by Sheridan et al., 2016. The gpPUL of *S. pneumoniae* R6 has been characterized by Marion et al., 2012. Other PULs are not reported in the literature and are identified according to the definition of PUL/gpPUL by searching their genomic assemblies. Genome accession numbers are presented at the top of each species. PL8: putative chondroitinase/hyaluronidase; KdgA: 2-keto-3-deoxygluconate aldolase: KdgK: 2-keto-3-deoxygluconate kinase; DhuI: 4-deoxy-L-threo-5-hexosulose-uronate ketol-isomerase; DhuD: 2-keto-3-deoxy-D-gluconate dehydrogenase; EIIA-EIID: Enzyme complex II subunit A to D of PTS system; PL12: putative heparinase; GalR: galactose operon repressor type transcription regulator; LaciR: lactose operon repressor type transcription regulator; GH27, GH43: glycoside hydrolase family 27 and 43 proteins; TCS: two-component response regulatory system; HK sensor: sensor protein of the Histidine kinase sensor system; ABC: ABC transporter proteins; Spi: sugar-phosphate isomerase; SGBP: surface glycan-binding protein; FBA: fructose biphosphate aldolase class II; Rpe: ribulose phosphate isomerase; BgiG: transcription anti-terminator; GntR: gluconate-operon repressor type transcription regulator; ΔGH: unsaturated glucuronyl hydrolase; Eda: Entner-Doudoroff aldolase: IclR: glyoxylate bypass repressor type transcription regulator; kduI: 5-dehydroxy-4deoxy-D-glucuronate Isomerase; RpiR: HTH-type transcriptional regulator; Sul: sulfatase; kduD: 5-dehydroxy-4deoxy-D-glucuronate dehydrogenase.

The GAG degradation is infrequently reported in the Non-Bacteroidetes gut bacteria. Consequently, the knowledge of genetic and enzymatic machinery required in intestinal Firmicutes and Proteobacteria for glycan degradation appears to lag behind that of *Bacteroides* by a significant margin. Nevertheless, efforts have been made to define the glycan utilization gene clusters in Firmicutes, termed as Gram-positive PULs (gpPULs), which are defined as a genetic loci that encode at least a carbohydrate degrading enzyme, a glycan transport system, and a transcription regulator.^115^ Unlike *Bacteroides*, the Firmicutes lack SusC homologs for internalization of degraded oligosaccharides.^115^ The regulation of distinct gpPULs is predicted to be regulated by a diverse number of transcription factors, many of which remain to be characterized. For GAG-specific gpPULs, a polysaccharide lyase gene must also be present in the gene cluster. Reports of such GAG gpPULs are scant. A putative gpPUL harboring PL12 heparin lyase has been reported in *Roseburia hominis* A2-183 *in silico* (Figure 6).^115^ This gpPUL also possesses an unsaturated glycoside hydrolase (ΔGH) gene, a TCS regulator gene, and three ABC transport system component genes. Similar gpPULs, targeting heparin, have been reported in the genomes of *Lactobacillus rhamnosus* Lc705 and ATCC 8530, *L. paracasei* JCM 8130, and *L. casei* ATCC 393 (Figure 6).^77^ gpPULs for chondroitin sulfate assimilation have not been reported in literature yet. However, in *S. intermedius*, a member of oral microbiota, the CS lyase and the sulfatase activities were found to be secreted in the media.^84^ The lysis activity was inducible and showed catabolite repression in the presence of glucose. By searching the genome, a putative gpPUL for GAGs degradation, comprising of a PL8 and PL12 gene, was found in *S. intermedius* NCTC 11324 (Figure 6). Putative PL8 containing gpPULs in other CS degrading Firmicutes (*H. hathewayi, E. faecium*, and *L. casei*) were also found in their genomes. In addition to PL8, these PULs also contain genes to encode transporter proteins (PTS or ABC transporter), glycoside hydrolase, and regulatory proteins (Figure 6). However, none of the gpPULs of gut Firmicutes have been characterized.

**Figure 7.**
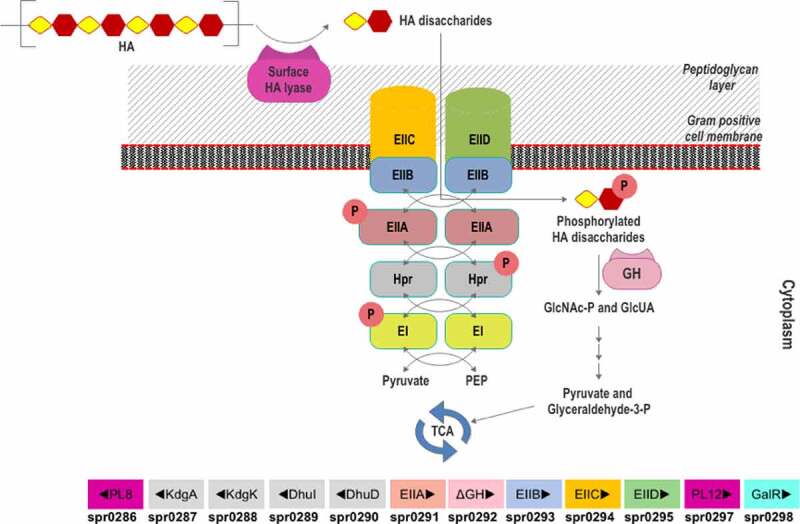
Scheme of HA utilization by *Streptococcus pneumoniae* R6. Extracellular HA is acquired and fragmented by a surface hyaluronidase to unsaturated HA disaccharides. Internalization of HAΔdi occurs *via* the phosphorylation-dephosphorylation cascade of PTS subunits (EI and EIIA-EIID), HPr protein, pyruvate, and phosphoenolpyruvate (PEP). The GlcNAc-phosphorylated HAΔdi is decomposed into monosaccharides (GlcA and GlcNAc-P) in the cytoplasm by an unsaturated glycoside hydrolase (GH). The monosaccharides are funneled through various enzymatic reactions to the energy production pathway. The HA-gpPUL with the gene numbers of the bacteria is shown at the bottom. PL8: hyaluronidase; KdgA: 2-keto-3-deoxygluconate aldolase: KdgK: 2-keto-3-deoxygluconate kinase; DhuI: 4-deoxy-L-threo-5-hexosulose-uronate ketol-isomerase; DhuD: 2-keto-3-deoxy-D-gluconate dehydrogenase; EIIA-EIID: Enzyme complex II subunit A to D of PTS system; PL12: putative heparinase; GalR: galactose operon repressor type transcription regulator; EI: Enzyme complex I of PTS system; Hpr: Hpr protein of PTS system.

Nevertheless, a gpPUL for HA utilization (HA-gpPUL) has been characterized in pathogenic Firmicutes of the *Streptococcal* genus.^116–118^ In *Streptococcus pneumoniae* R6, this HA-gpPUL contains genes encoding a hyaluronidase, an ΔGH, phosphotransferase system (PTS) transporter proteins, a regulatory protein, and the proteins required for downstream catabolism of monosaccharides and a putative heparinase gene (Figure 6). A typical HA degradation pathway in *S. pneumoniae* R6 strain is shown in Figure 7. A hyaluronidase-PL8 is present either on the cell surface or extracellularly, which decomposes HA to produce unsaturated HA disaccharides (HAΔdi).^117^ These disaccharides are internalized to the cytoplasm by the phosphotransferase system (PTS) and are phosphorylated at GlcNAc during the process. In the cytoplasm, ΔGH catabolizes the phosphorylated HAΔdi to produce GlcUA and phosphorylated GlcNAc. GlcUA, by a series of reactions, enters the TCA cycle to complete the HA breakdown. Although a putative transcription regulator (GalR) is present in the *Streptococcus* HA-gpPUL, it is not yet verified to be functional.

*Proteus vulgaris*, a Proteobacteria frequently found in the human gut, possesses two well-known chondroitin ABC lyases (ChS ABC I and ChS ABC II).^87,88^ Similar to *S. intermedius*, the chondroitin lyase activity of *P. vulgaris* is inducible by CS and repressed by glucose and other glycolytic intermediates.^119^ The two characterized chondroitin ABC lyases of *P. vulgaris* were shown to act on a wide range of substrates, including CS, DS, HA, and CSPG.^86^ The ChS ABC I lyase is an endolyase and is more active on polymeric CS. In contrast, the ChS ABC II lyase displays an exolytic mode of action, with higher processivity toward CS oligosaccharides. ChS ABC I lyase might first act on the polymeric CS, followed by ABC II lyase on the oligosaccharides in the gut environment. The two CS lyase genes of *P. vulgaris* were reported to be present in the same operon, which also consists of a putative sulfatase gene, a ΔGH, putative PTS transporter genes, and the genes required for monosaccharide catabolism (Figure 6).^120,121^ CS depolymerization seems to occur before desulfation.^122^ As the CS lysis activity was inducible by GalNAc, the monosaccharide end product might act as an activator.^119^ Whether the sulfated CS disaccharide activates the regulator remains unknown.

## Prospects and conclusion

Colonic GAGs provide an abundant source of nutrition and colonization factors for diverse HGM members. In addition, dietary and medicinal (oral) administration of GAGs is reported to modulate the HGM composition. The present review summarizes the reported GAG-degraders and their enzymatic pathway for GAG utilization in various HGM phyla. At present, the information on HGM-involved degradation of GAGs is highly skewed toward *Bacteroides*. In contrast, the GAG-degradation pathways in bacteria of other HGM phyla (Firmicutes, Proteobacteria, and Actinobacteria) are not extensively studied, although several GAG degraders from these phyla have been isolated. Especially, Firmicutes represents the largest phylum of gut microbiota, and several Firmicutes from the gut have shown HA, CS, and heparin degradation capabilities. Various members of gut Firmicutes have been shown to be beneficial for the host as well as essential for the HGM homeostasis. Specific details of the GAG utilization by the gut Firmicutes would augment our understanding of its effects on human health. Though the number and diversity of Proteobacteria are less than Firmicutes and *Bacteroides*, they represent the major group implicated in opportunistic pathogenesis and show rapid overgrowth during gut dysbiosis. The systemic details of Proteobacterial GAG degradation could assist in negating the ill-effects of pathogenic disorders caused by these bacteria. In addition, as GAGs are indigestible in the human intestine, the therapeutic/prebiotic effects of oral GAGs could arise from the HGM-derived catabolism. A thorough understanding of the differences in the GAG utilization mechanisms between different HGM phyla could explain the modulatory effects of prebiotic GAGs on HGM. Continuous isolation of gut bacteria, which can degrade GAGs and provide beneficial effects, will improve the application of modulating intestinal health. Transcriptomic studies can be effectively used to identify GAG degrading enzymatic pathways. Biochemically, characterizing the activity and substrate specificity of PLs, sulfatases, and other proteins is critical for elucidating the precise utilization pathway of GAGs by various gut bacteria. It is also critical to evaluate the contribution of GAG utilization for the colonization of gut bacteria.

Efforts have been made to modify the HGM of individuals for medical purposes. For example, fecal transplants from healthy individuals have been administered, as treatment, in patients suffering from gastrointestinal, metabolic, immunological, and neurological disorders.^123^ However, the success of the treatment depends on the effective colonization of the transplanted fecal bacteria. Previously, it has been shown that several bacterial species across various phyla utilize GAGs to colonize the gut microenvironment. In addition, numerous fecal transplant studies in humans and animals generally show an increase in the abundance of GAG-degrading bacteria such as various *Bacteroides* strains, *Clostridium* cluster IV (contains *F. prausnitzii), Clostridium* cluster XIVa (contains *Hungatella* species), *Enterococci*, and *Lactobacilli*.^123^ Therefore, it would be valuable to ascertain the contribution of GAG utilization of the specific isolated members for their successful colonization. Formulating a stable artificial gut micro-community by combining these isolates holds the potential to promote transplant applications.

Additionally, pathogenic bacteria can colonize the gut tissues using GAGs and PGs as the site of entry and a source of nutrition.^19^ For example, enteric pathogens like *Toxoplasmosis gondii, E. coli* O157:H7,^19^ and various opportunistic pathogenic *Streptococci*^124^ are reported to use GAGs as the entry point for infecting the intestinal cells. Currently used antibiotic regimens against gastrointestinal pathogens also affect the mutualistic bacteria, which can cause gut dysbiosis and impair the normal functioning of the HGM. A detailed study of the mechanistic differences in GAG utilization between pathogens and gut commensals could assist in designing better antibacterial strategies which can remove the pathogenic bacteria without impacting the HGM.
